# The role of adenosine diphosphate mediated platelet responsiveness for the stability of platelet integrity in citrated whole blood under *ex vivo* conditions

**DOI:** 10.1371/journal.pone.0188193

**Published:** 2017-11-20

**Authors:** Juergen Koessler, Michaela Schwarz, Katja Weber, Julia Etzel, Angela Koessler, Markus Boeck, Anna Kobsar

**Affiliations:** Institute of Transfusion Medicine and Haemotherapy, University of Wuerzburg, Wuerzburg, Germany; Royal College of Surgeons in Ireland, IRELAND

## Abstract

**Background:**

Platelets are important for effective hemostasis and considered to be involved in pathophysiological processes, e.g. in cardiovascular diseases. Platelets provided for research or for therapeutic use are frequently separated from citrated whole blood (WB) stored for different periods of time. Although functionally intact platelets are required, the stability of platelet integrity, e.g. adenosine diphosphate (ADP) mediated responsiveness, has never been thoroughly investigated in citrated WB under *ex vivo* conditions.

**Objectives:**

Platelet integrity was evaluated at different time points in citrated WB units, collected from healthy donors and stored for 5 days at ambient temperature. The analysis included the measurement of activation markers, of induced light transmission aggregometry and of purinergic receptor expression or function. Inhibitory pathways were explored by determination of basal vasodilator-stimulated phosphoprotein (VASP)-phosphorylation, intracellular cyclic nucleotide levels and the content of phosphodiesterase 5A. Fresh peripheral blood (PB) samples served as controls.

**Results:**

On day 5 of storage, thrombin receptor activating peptide-6 (TRAP-6) stimulated CD62P expression and fibrinogen binding were comparable to PB samples. ADP induced aggregation continuously decreased during storage. Purinergic receptor expression remained unchanged, whereas the P2Y1 activity progressively declined in contrast to preserved P2Y12 and P2X1 function. Inhibitory pathways were unaffected except for a slight elevation of VASP phosphorylation at Ser^239^ on day 5.

**Conclusion:**

After 5 days of storage in citrated WB, platelet responsiveness to TRAP-6 is sufficiently maintained. However, ADP-mediated platelet integrity is more sensitive to deterioration, especially after storage for more than 2 days. Decreasing ADP-induced aggregation is particularly caused by the impairment of the purinergic receptor P2Y1 activity. These characteristics should be considered in the use of platelets from stored citrated WB for experimental or therapeutic issues.

## Introduction

Platelets are anucleated cell fragments derived from megakaryocytes and play an important physiological role in hemostasis, wound healing or inflammation [1,2]. There is also growing evidence that platelets are involved in pathophysiological processes like atherosclerosis, diabetes or cancer.

The analysis of molecular mechanisms in platelets is, therefore, an interesting field of pharmacological or experimental research, e.g. addressing cardiovascular or hemostatic questions. For this purpose, it is crucial to provide platelets with maintained functionality to achieve reliable results. Blood specimens for experimental studies are commonly collected from healthy volunteers as whole blood (WB) anticoagulated with citrate solutions. However, the preparation of platelets and many analytical procedures are laborious and time-consuming. Therefore, it would be advantageous to store blood samples *ex vivo* for a certain period of time before further processing, but the preservation of platelet function has to be warranted. In transfusion medicine, WB units are not only an essential starting product for the preparation of blood components. There is an ongoing debate on the direct utilization of citrated WB, referred to as “warm blood”, for acute hemorrhage, e.g. in the military setting [3–5]. “Warm blood” is assumed to mediate potent hemostasis within 48 h after collection [5]. However, the contribution of functional platelets to the hemostatic capacity of these blood products has not entirely been investigated.

In this context, the proper responsiveness of platelets to adenosine diphosphate (ADP) is a significant characteristic of platelet integrity. In contrary, the inhibition of ADP induced aggregation is a major pharmacological principle for the treatment of cardiovascular diseases, e.g. after stent implantation in coronary heart disease, and is associated with an increased bleeding risk.

ADP exerts its effects via purinergic platelet receptors: P2Y1, P2Y12 and P2X1. The receptors P2Y1 and P2Y12 are guanine nucleotide-binding-protein (G-protein) coupled receptors [6–8], whereas P2X1 is an adenosine triphosphate (ATP)-gated, non-selective cation channel. P2Y1 is a G_q_-coupled receptor, activating platelet phospholipase C and stimulating calcium release from intracellular stores [7,9]. P2Y12 inhibits platelet adenylyl cylase through G_αi_ [7,9]. Simultaneous activation of both P2Y1 and P2Y12 results in platelet aggregation. Stimulation of the P2X1 receptor alone causes a rapid calcium influx in platelets that can synergize P2Y1 effects [10] and induce platelet shape change, but is not able to induce platelet aggregation [11].

Recently, we investigated the deterioration of ADP responsiveness in washed platelets, which are often required for experimental studies. We could show that ADP induced aggregation decreases rapidly after the washing procedure within 30 min, which is particularly caused by impaired activity of the P2Y1 receptor associated with disturbed calcium regulation [12].

In this study, we intended to elucidate the course of ADP-mediated platelet integrity under *ex vivo* conditions in citrated WB stored for a time period of 5 days at ambient temperature, addressing aggregometry, purinergic receptor expression and purinergic receptor activity. The results render novel insight into changes of platelet integrity under *ex vivo* conditions, important for reasonable handling of blood samples in experimental medicine and for blood banking practices in transfusion medicine.

## Materials and methods

### Materials

ADP was from Haemochrom Diagnostica GmbH (Essen, Germany), thrombin receptor activating peptide-6 (TRAP-6) was obtained from BACHEM (Weil am Rhein, Germany). Mouse monoclonal FITC-conjugated anti-fibrinogen antibody was from STAGO Germany (Düsseldorf, Germany), mouse monoclonal FITC-conjugated anti-CD62P antibody was from Acris antibodies GmbH (Herford, Deutschland). APC-conjugated mouse anti-human CD41a antibody was from BG Biosciences (Heidelberg, Germany). FITC-conjugated goat anti-rabbit polyclonal antibody, prostaglandin E1 (PGE1), acetylsalicylic acid (ASS), probenecid, Pluronic F-127, 4-[2-hydroxyethyl]-1-piperazineethanesulfonic acid (HEPES) and apyrase were from Sigma-Aldrich Chemie GmbH (Muenchen, Germany). Rabbit polyclonal anti-P2Y1, anti-P2Y12 and anti-P2X1 antibodies were from Alomone Labs (Jerusalem, Israel). The selective P2Y1 receptor agonist [(1*R*,2*R*,3*S*,4*R*,5*S*)-4-[6-Amino-2-(methylthio)-9*H*-purin-9-yl]-2,3-dihydroxy- bicycle [3.1.0] hex-1-yl]methyl] diphosphoric acid mono ester trisodium salt (MRS2365), the selective antagonist of P2Y1(1R*,2S*)-4-[2-Iodo-6-(methylamino)-9*H*-purin-9-yl]-2-(phosphornooxy)bicyclo-[3.1.0]hexane-1-methanol dihydrogen phosphate ester tetraammonium salt (MRS2500), the agonist of P2X1 receptor α,β-Methyleneadenosine 5'-triphosphate trisodium salt (α,β-MeATP), and the potent P2X1 antagonist 4,4',4'',4‴-[Carbonylbis(imino-5,1,3-benzenetriyl-*bis*(carbonylimino))] *tetrakis*-1,3-benzenedisul—fonic acid, octasodium salt (NF449) were from R&D Systems GmbH (Wiesbaden-Nordenstadt Germany). Fluo-4A M Cell permeant was from Life Technologies GmbH (Darmstadt, Germany). Flow cytometric PLT VASP/P2Y12 Kit for the measurement of P2Y12 receptor function was from STAGO GmbH (Duesseldorf, Germany). Rabbit polyclonal anti-PDE5A antibody and rabbit monoclonal anti-pan-actin antibody were from New England Biolabs GmbH (Frankfurt am Main, Germany). Mouse monoclonal phospho-VASP Ser^239^ and phospho-VASP Ser^157^ antibodies were from Nanotools (Teningen, Germany). Horseradish peroxidase-conjugated goat anti-rabbit and anti-mouse antibodies were from Bio-Rad Laboratories, Inc. (München, Germany).

### Blood collection

Venous PB samples and WB units were obtained from informed healthy voluntary donors (aged from 19 to 42 years, 7 male, 6 female, without any medication 14 days before donation) in the blood donation department of our institute between February and August 2015. The participants had to meet the official criteria for blood donations in Germany for inclusion. The WB units were stored for five days under continuous agitation on a flatbed shaker at ambient temperature (22 ± 2°C).

Freshly obtained PB samples served as controls. They were collected in polystyrene tubes containing 3.2% citrate buffer (106 mM trisodium citrate, Sarstedt, Nuembrecht, Germany) and analysis started within 1 h.

WB units were collected in polyvinylchloride (PVC) bags (Haemonetics, Bothwell, Scotland, UK), containing 70 mL of citrate-phosphate-dextrose solution, CPD [0.327 g citric acid (monohydrate), 2.63 g sodium citrate (dihydrate), 0.222 g monobasic sodium phosphate (monohydrate), 2.55 g glucose (monohydrate), aqua ad iniectabilia ad 100 mL], using the blood mixing and weighing device Topswing Pro (Transmed Medizintechnik GmbH & Co. KG, Bad Wuennenberg, Germany) within maximal 12 min. The collection bag was separated from the lines by welds directly at the end of donation. For analysis, samples from WB units were taken under sterile conditions at the following time points: at day 0 (16–20 h), at day 2 and at day 5 after donation.

Our studies with human platelets and the consent procedure were approved by the local ethics committee of the University of Wuerzburg (approval number 47/12). All participants provided their written informed consent. The study was performed according to our institutional guidelines and to the Declaration of Helsinki.

### Measurement of metabolic parameters

Lactate, glucose, hemoglobin or potassium concentrations and pH were measured with the blood gas analyzer ABL800 basic from Radiometer GmbH (Willich, Germany), blood cell counts with the hematology analyzer KX21N from Sysmex GmbH (Norderstedt, Germany).

### Preparation of washed platelets from PB

Washed platelets were prepared as described [13]. 3 mM EGTA were added to PB to prevent platelet activation. Platelet-rich plasma (PRP) was obtained by centrifugation of PB at 330 g for 5 minutes (min). Subsequently, samples of PRP were centrifuged at 430 g for 10 min. Then pelleted platelets were washed once in CGS buffer (120 mM sodium chloride, 12.9 mM trisodium citrate, 30 mM D-glucose, pH 6.5) and re-suspended in HEPES buffer (150 mM NaCl, 5 mM KCl, 1 mM MgCl_2_, 10 mM D-glucose, 10 mM HEPES, pH 7.4) to the final appropriate concentration. After resting for 15 min in a 37°C water bath, washed platelets were used for the measurement of cyclic nucleotides and for Western blot experiments. For experiments with platelet stimulation, CaCl_2_ was added in a final concentration of 1μM to the suspension of washed platelets shortly before stimulation.

### Preparation of washed platelets from stored WB units

40 mL of stored WB were diluted with 10 mL PBS, and EGTA was added to a final concentration of 3 mM to prevent platelet activation. This mixture was centrifuged for 14 min at 330 g. The supernatant (diluted PRP) was diluted 1:1 with PBS one more time and centrifuged for 10 min at 160 g to precipitate residual erythrocytes. The erythrocyte-free supernatant was transferred into a new tube and centrifuged for 15 min at 430 g. The pellet was washed in 5 mL of CGS buffer and re-suspended in HEPES buffer as described above. Washed platelets were used for the measurement of cyclic nucleotides and for Western blot experiments. For platelet stimulation, CaCl_2_ was added in a final concentration of 1μM as described above.

### Platelet preparation for the measurement of P2Y1 activity

To prepare platelets for P2Y1 activity measurement, PGE1 (500 nM final concentration) was added to PRP or stored WB (as described for the preparation of washed platelets) and then centrifuged at 430 g for 10 min. The pellet was washed with 5 mL of modified Tyrode buffer (10 mM HEPES, 150 mM NaCl, 3 mM KCl, 1 mM MgCl_2_, 5 mM glucose and 0.1% BSA, pH 6.5) containing 500 nM PGE1. Platelets were resuspended in modified Tyrode buffer without PGE1 and platelet concentration was adjusted to 0.6 x10^8^ platelets/mL [14].

### Platelet preparation for the measurement of P2X1 activity

To prepare platelets for P2X1 activity measurement, ASS (1 mM) and apyrase (0.3 U/mL) were added to the PRP or stored WB (as described for the preparation of washed platelets) and then centrifuged at 430 g for 10 min. The pellet was washed with 5 mL of modified Tyrode buffer containing 1 mM ASS and 0.3 U/mL apyrase. Platelets were resuspended in modified Tyrode buffer containing 0.3 U/mL apyrase and platelet concentration was adjusted to 0.6 x10^8^ platelets/mL [14].

### Measurement of P2Y1 and P2X1 activity

The activity of platelet purinergic P2Y1 and P2X1 receptors was measured by calcium flux induced fluorescence in Fluo-4AM loaded platelets after selective stimulation as described [14]. Briefly, in each well of a 96-well black plate, 100 μL of washed platelets were mixed with an equal volume of Hank’s buffered saline solution (HBSS) containing 10 mM HEPES, 0.1% BSA, 2.5 mM probenecid, 1 mM EGTA, 0.01% pluronic acid and 2 μM Fluo-4AM at pH 7.4. For P2X1 measurements, EGTA was substituted by 2.5 mM calcium and apyrase was added in a final concentration of 0.3 U/mL. The plate was incubated for 20 min at room temperature in the dark, followed by 20 min incubation at 37°C. During the last 10 min of incubation, 2 μL of 100 μM MRS2500, a P2Y1 antagonist, or 2 μL of 100 μM NF449, a P2X1 antagonist, were added. After measurement of the basal fluorescence (Ex 488—Em 538; 20 measurements at 1 second), platelets were stimulated with 2 μL of 100 μM MRS2365, a P2Y1 agonist, or 2 μL of 100 μM α,β-MeATP, a P2X1 agonist. After stimulation, fluorescence values were measured every second for the next 3 min. Fluorescence signals were measured and analyzed by Fluoroscan Ascent Microplate Fluorometer from Fisher Scientific GmbH (Schwerte, Germany).

### Measurement of P2Y12 activity

The activity of platelet P2Y12 receptor was measured by the flow cytometric PLT VASP/P2Y12 Kit. Briefly, aliquots of PB or WB diluted with plasma to 3x10^8^ platelets/mL were stimulated with PGE1 alone or with a combination of PGE1 and ADP at RT. After stimulation, samples were fixed and stained as described in the manufacturer's instructions, followed by flow cytometric measurement of fluorescence. Platelet reactivity index (PRI) was calculated using corrected mean fluorescence intensities (MFIc) as PRI = [MFIc (PGE1)–MFIc (PGE1 + ADP)] / [MFIc (PGE1)] x 100%.

### Protein concentration

The protein concentration of samples was measured with BCA Protein Assay Reagent following the manufacturer's instructions (Thermo Fisher Scientific p/a Perbio Science German, Bonn, Germany).

### Flow cytometric analysis of platelets from stored WB units

For the measurement of CD62P expression on platelets from PB and stored WB units, 15 μL of PB or WB diluted with 15 μL PBS were pre-incubated with 6 μL APC-conjugated mouse anti-CD41a and 10 μL FITC-conjugated anti-CD62P antibodies for 10 min at ambient temperature in the dark and then stimulated for 2 min with 10 μM TRAP-6 or with PBS as control, respectively. For the measurement of fibrinogen binding on platelets, 15 μL of PB or stored WB were pre-incubated with 15 μL of FITC-conjugated mouse anti-fibrinogen antibody and 6 μL of allophycocyanin-conjugated mouse anti-CD41a antibody for 10 min at ambient temperature in the dark and then stimulated for 2 min with 10 μM TRAP-6 or with PBS as control, respectively. Reactions were stopped with 0.1% formaldehyde, samples were diluted with 400 μL PBS/5 mM glucose/0.5% BSA and subsequently analyzed by flow cytometry as described above.

### Platelet aggregation

Platelet aggregation was measured using an APACT 4004 aggregometer (LabiTec, Ahrensburg, Germany). PRP from PB or from stored WB units were stimulated with 10 μM TRAP-6 or 10 μM ADP. Aggregation was measured for 5 min under continuous stirring at 1,000 rpm and 37°C.

### cAMP and cGMP measurement

For cAMP and cGMP measurements, the platelet concentration of washed platelets was adjusted to 3 x 10^8^ platelets per mL. Samples were lysed with 5% trichloroacetic acid. After extraction with ether, cAMP and cGMP were detected by cAMP EIA (enzyme-linked immunoassay) Kit and GMP EIA Kit, respectively, following the manufacturer's instructions (Cayman Chemical, Hamburg, Germany).

### Western blot analysis

Cell lysates of washed platelets containing 2 μg protein were loaded onto the gel, separated by SDS-PAGE and then transferred onto nitrocellulose membranes. The membranes were incubated with appropriate primary antibodies overnight at 4°C. For visualization of the signal, goat anti-rabbit or anti-mouse IgG conjugated with horseradish peroxidase were used as secondary antibodies, followed by detection with an ECL detection kit (GE Healthcare, Piscataway, NJ, USA). Blots were analyzed densitometrically using NIH Image J software (http://rsbweb.nih.gov/ij/; National Institutes of Health, Bethesda, MD, USA) for uncalibrated optical density.

### Statistical analysis

Data are presented as mean ± standard error of the mean (SEM). The n-values refer to the number of experiments, each made with different blood donors. Differences between groups were analyzed by paired and un-paired Student's t-test as appropriate using MedCalc statistic program (MedCalc Software bvba, Mariakerke, Belgium). P<0.05 was considered statistically significant.

## Results

### Cellular and metabolic characteristics of citrated WB during storage

The basic characteristics of citrated WB after collection on day 0 represent values as expected for healthy donors (Table 1). The obvious elevation of platelet count on day 5 may be caused by interferences due to morphological changes of stored blood cells, accompanied by a slight tendency of reduction for red cells and leukocytes. The pH values in WB units decreased associated with continuous glucose consumption, whereas lactate and potassium concentrations increased, achieving almost 4 and 2.7 fold levels compared to day 0.

**Table 1:** Basic characteristics of PB samples and WB units during storage.

Parameter	unit	PB	day 0	day 2	day 5
platelets	x10^3^	207±12	167 ± 15	172 ± 17	229 ± 20 *^#^
red cells	x10^6^	4.7±0.2	4.1 ± 0.3	3.8 ± 0.2	3.7 ± 0.1
leukocytes	x10^3^	4.8±0.4	5.4 ± 0.4	5.6 ± 0.3	4.2 ± 0.3 ^#^
hemoglobin	g/dL	13.9±0.3	12.5 ± 0.6	11.5 ± 0.6	11.0 ± 0.3
hematocrit	%	40.7±1.5	38.4 ± 2.8	35.9 ± 2.2	35.1 ± 1.0
pH	-	7.51±0.02	7.08 ± 0.01	6.92 ± 0.02 *	6.60 ± 0.08 *^#^
lactate	mmol/L	1.8±0.2	5.6 ± 0.1	10.0 ± 0.9 *	22.0 ± 2.1 *^#^
glucose	mg/dL	80±8	349 ± 5	302 ± 12 *	191 ± 19 *^#^
potassium	mmol/L	3.3±0.3	3.5 ± 0.2	4.8 ± 0.2 *	9.4 ± 0.1 *^#^

* P < 0.05, compared to day 0

^#^ P < 0.05, compared to day 2

mean ± SEM; n = 3.

In PB samples, collected in tubes with a different anticoagulant medium, the results were also according to values for healthy individuals measured at 22° C (Table 1).

### Platelets in citrated WB show minor pre-activation and maintained responsiveness during storage for 5 days

**Fig 1:**
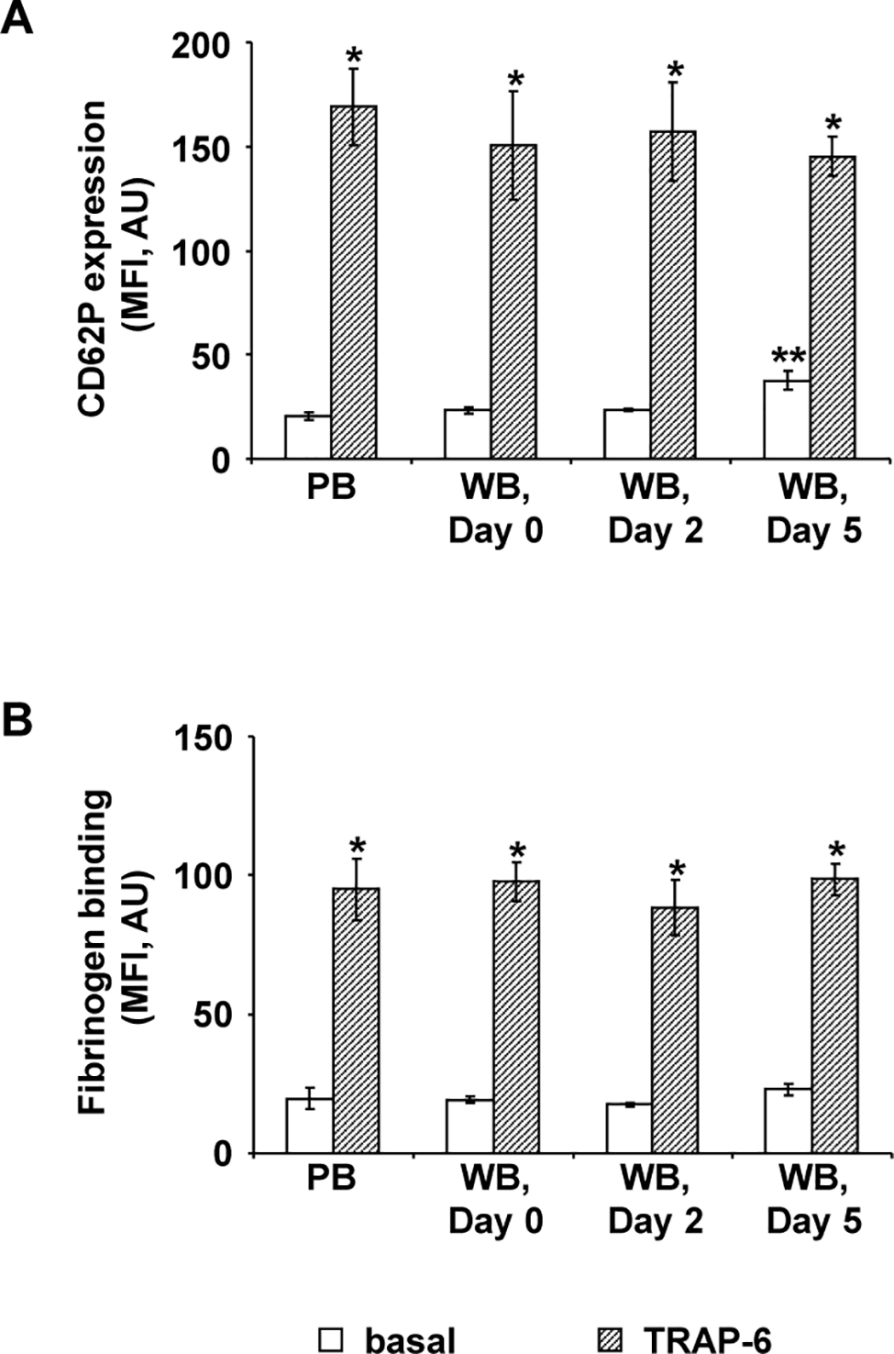
Time-dependent CD62P surface expression and fibrinogen binding of platelets in citrated WB. The histograms show the mean fluorescence intensities (MFI) of basal (white columns) and 10 μM TRAP-6 stimulated (shaded columns) CD62P expression (A) or fibrinogen binding (B) in fresh PB and in stored WB units at different time points as indicated. Results are presented in absolute arbitrary units (AU) as mean fluorescence ± SEM; n = 5; *: p < 0.05 (stimulated values compared to basal values); **: p < 0.05 (compared to PB and to WB at day 0).

Pre-activation of platelets was investigated by basal CD62P expression and basal fibrinogen binding (Fig 1). Initially, the basal platelet CD62P expression was comparable in PB samples and in WB units on day 0 with 20.5±2.0 AU and 23.4±1.6 AU respectively (Fig 1A). Under storage, it was unchanged after 2 days, but slightly increased with 37.6±4.5 AU on day 5 (Fig 1A). In fresh PB, basal fibrinogen binding was 19.7±3.7 AU and not different in WB units stored for 5 days (Fig 1B).

10 μM TRAP-6 stimulation increased CD62P expression and fibrinogen binding approximately up to six and four fold respectively, with comparable values in PRP from fresh PB samples and from WB units at all time points (Fig 1A and 1B).

### ADP induced aggregation continuously decreases in citrated WB.

**Fig 2:**
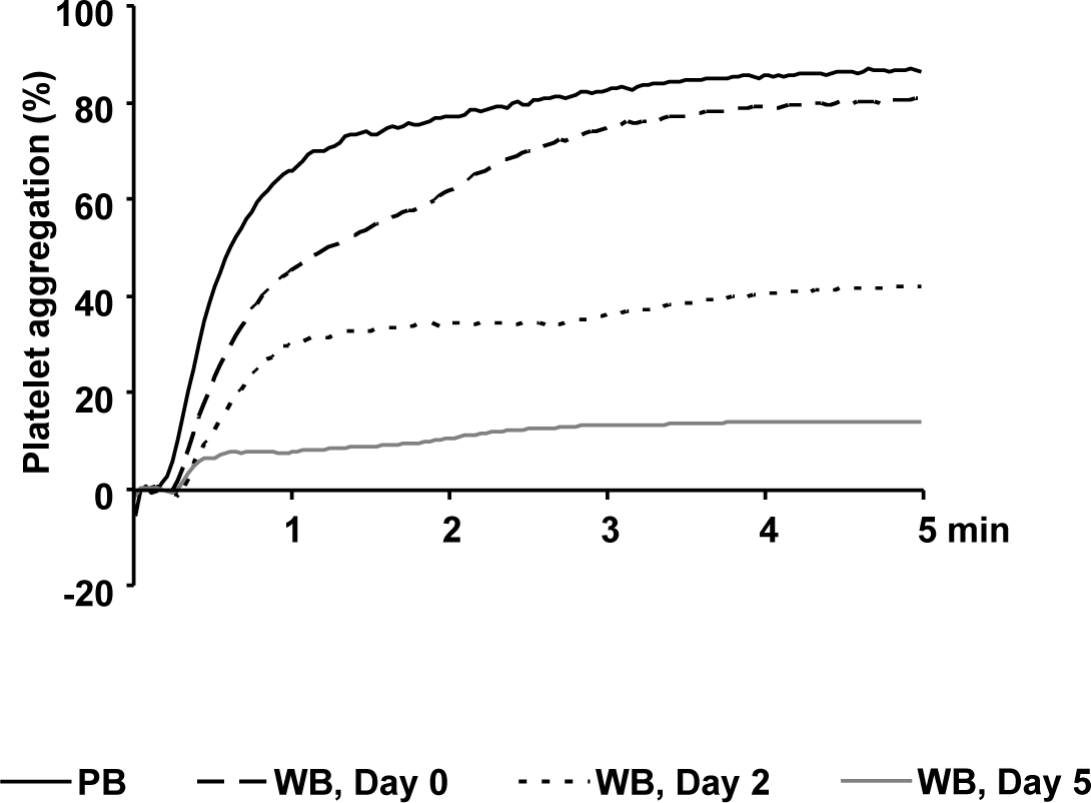
ADP induced platelet aggregation continually decreases in citrated WB. Light transmission aggregometry induced with 10 μM ADP was measured in PRP from PB samples and from stored WB units at different time points as indicated. Mean aggregation traces are presented; n = 5.

Light transmission aggregation induced by 10 μM ADP reached values above 80% in PRP from fresh PB samples (Fig 2). PRP from WB units showed slightly attenuated aggregation levels with 80.8±4.6% on day 0 compared to PRP from fresh PB. The aggregation responses developed a partial reduction on day 2 with 41.8±19.4% and a near-total decrease on day 5. Aggregation induced by 10 μM TRAP-6 was preserved for the storage period of 5 days with values above 80% (data not shown).

### Purinergic receptor expression remains unchanged in citrated WB for 5 days

**Fig 3:**
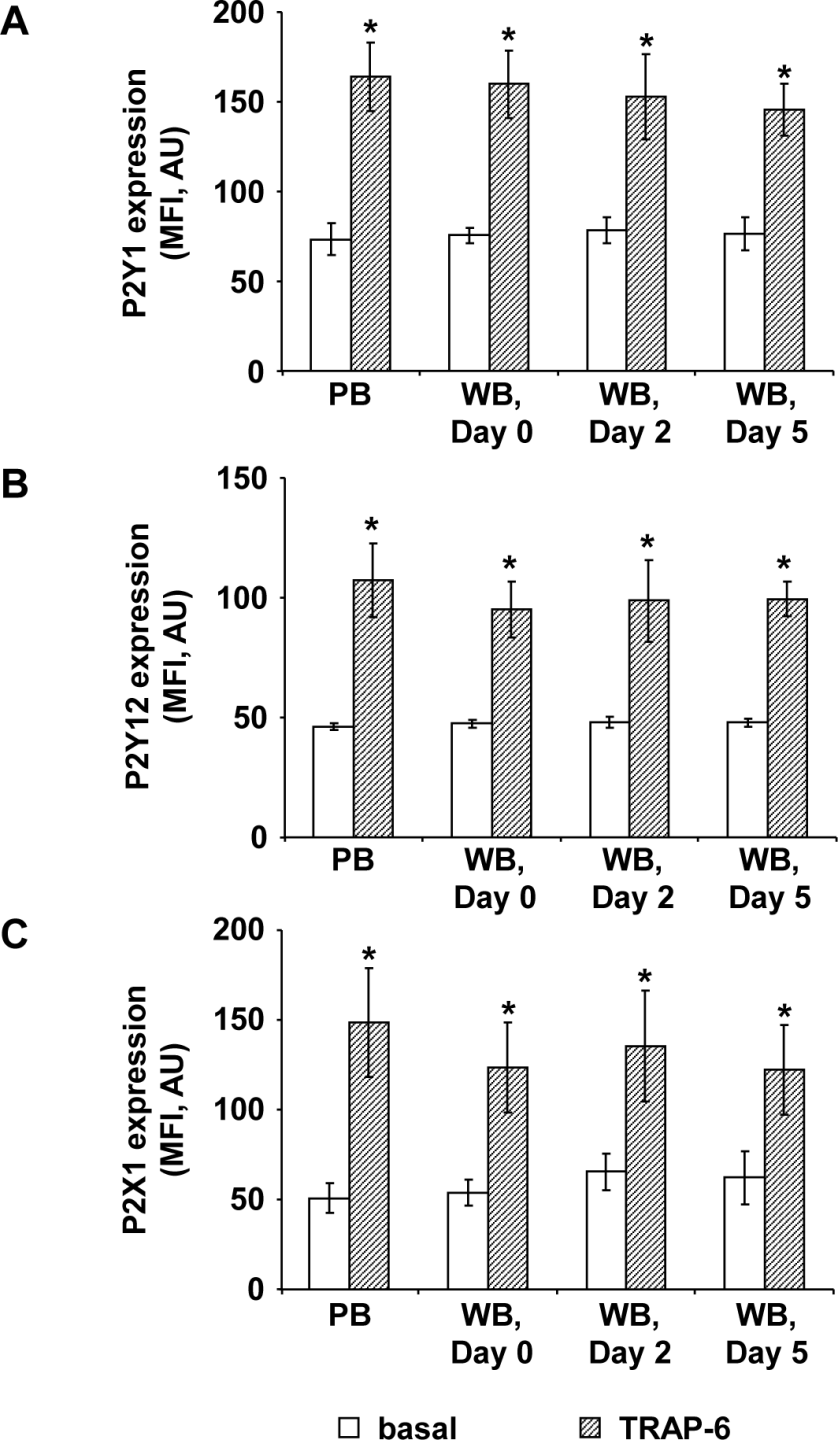
Platelet purinergic receptor expression in citrated WB is maintained during storage. The histograms show the mean fluorescence intensities (MFI) of basal (white columns) and 10 μM TRAP-6 stimulated (shaded columns) P2Y1- (A), P2Y12- (B), and P2X1- (C) receptor expression in fresh PB samples and in stored WB units at different time points as indicated. Results are presented in absolute arbitrary units (AU) as mean fluorescence ± SEM; n = 6; *: p < 0.05 (compared to basal values).

The basal expression of platelet purinergic receptors (P2Y1, P2Y12, and P2X1) in freshly prepared WB units did not differ from PB and remained preserved during storage for 5 days (Fig 3). Stimulation of fresh PB with 10 μM TRAP-6 resulted in an approximately two fold increase of expression for all investigated purinergic receptors, comparable to platelets from fresh or stored WB units (Fig 3).

### The P2Y1 receptor activity continuously decreases in citrated WB

**Fig 4:**
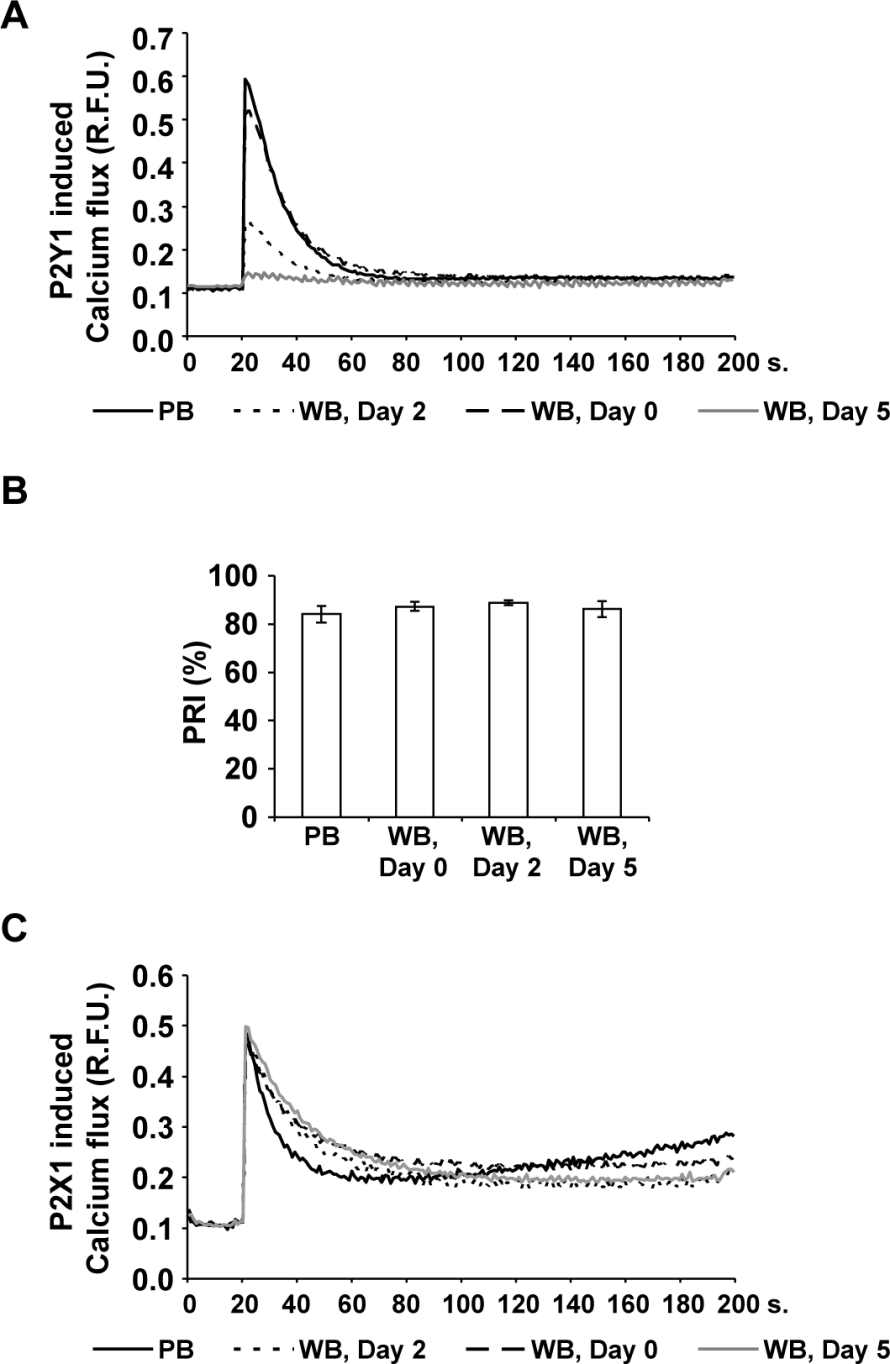
The activity of platelet P2Y1, but not of P2X1 or P2Y12 receptor, continually decreases in citrated WB. Calcium induced fluorescence curves were generated with Fluo-4AM loaded freshly washed platelets from PB samples (black line) and from WB units on Day 0 (dashed line), on Day 2 (dotted line) and on Day 5 (gray line) after stimulation with the P2Y1 agonist MRS2365 (A) or the P2X1 agonist α,β-MeATP (C). Mean fluorescence curves are presented (expressed in relative fluorescence units, R.F.U.); n = 6. For the P2Y12 receptor activity, mean PRI values (B) were determined in PB samples and in stored WB units at different time points as indicated. Results are presented as mean % (PRI) ± SEM; n = 4.

The maximal P2Y1-induced calcium flux reached 0.59±0.08 R.F.U. in platelets from fresh PB and, similarly, 0.51±0.06 R.F.U. in freshly prepared WB units (Fig 4A). Under storage, it decreased by 56% to 0.26±0.03 R.F.U on day 2 and by 76% to 0.14±0.02 R.F.U. on day 5.

The purinergic receptor P2Y12 activity was characterized by PRI determination (Fig 4B), which was comparable in PB and WB samples at all time points with values of more than 80%. Similar to P2Y12, the activity of the purinergic receptor P2X1 showed consistent results in PB and in WB units throughout storage (Fig 4C).

### Platelet inhibitory signaling remains unaffected except for a slight increase of VASP phosphorylation at Ser^239^

Since the P2Y12 receptor function is associated with the inhibitory signaling system via the adenylyl cyclase activity, the basal VASP phosphorylation, the cyclic nucleotide levels and the content of PDE5A, as the specific cGMP degrading enzyme, were additionally determined.

**Fig 5:**
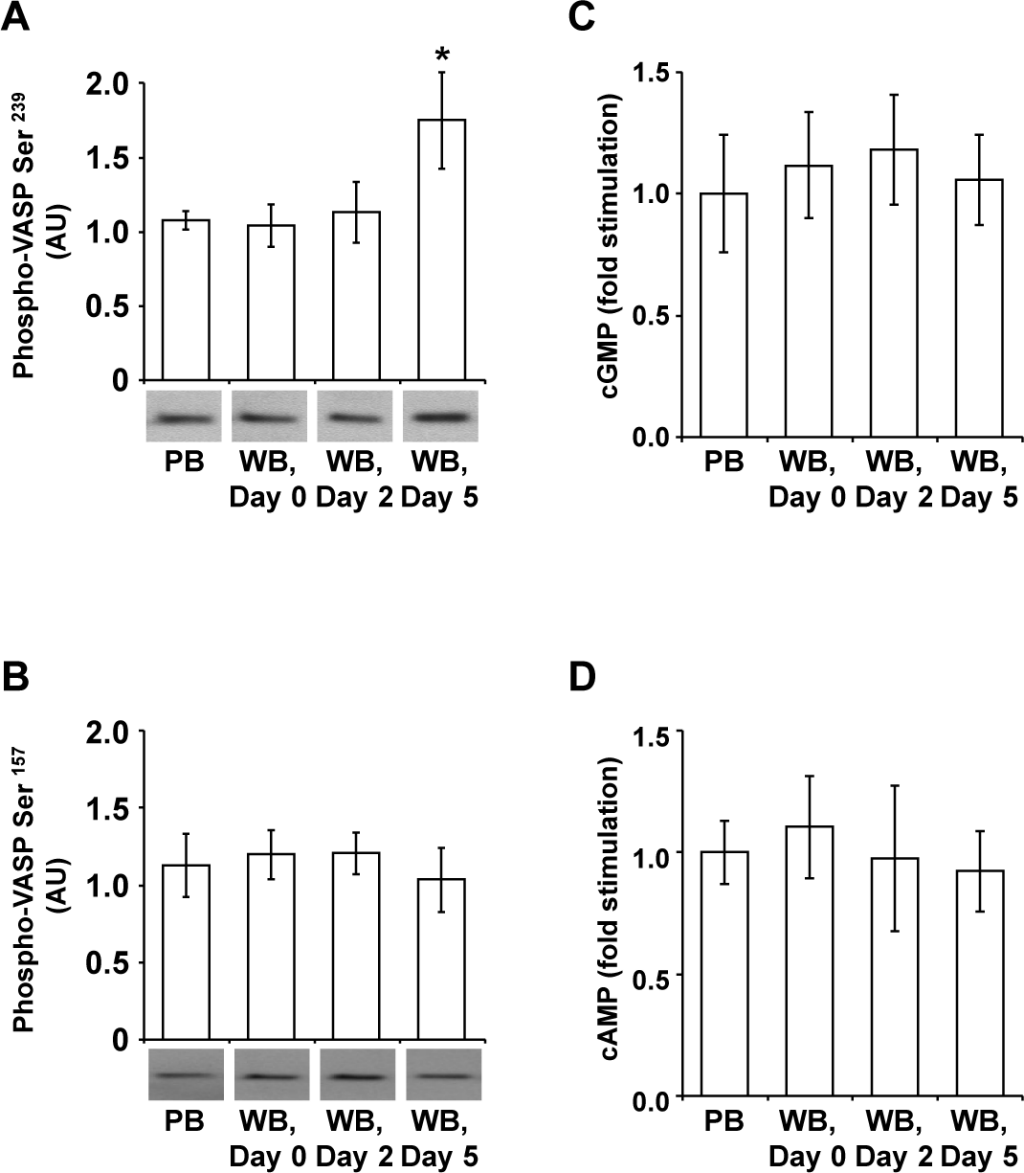
Basal VASP phosphorylation and basal cyclic nucleotide levels in citrated WB. Quiescent washed platelets (3 x 10^8^ per mL) from fresh PB samples and from stored WB units were lysed with Laemmli buffer and analyzed on Western blot for VASP Ser^239^ (A) and VASP Ser^157^ (B) phosphorylation. After scanning bands were quantified by the Image J program. Results are presented in arbitrary units (AU) as mean ± SEM; n = 8; *: p < 0.05 (compared to values of PB and of WB units on day 0). Cyclic nucleotide concentrations were measured in lysed washed platelets (3 x 10^8^ per mL) from fresh PB samples and from stored WB units. After extraction with ether the contents of cGMP (C) and cAMP (D) were determined with immunoassays. Results are presented as mean (fold stimulation) ± SEM; n = 8; *: p < 0.05 (compared to basal values of PB and of stored WB units on day 0).

The basal VASP phosphorylation levels at Ser^157^ and at Ser^239^ were similar in PB samples and in WB units (Fig 5A and 5B). Only on day 5, there was a slight elevation by approximately 1.6 fold at Ser^239^ (Fig 5B).

**Fig 6:**
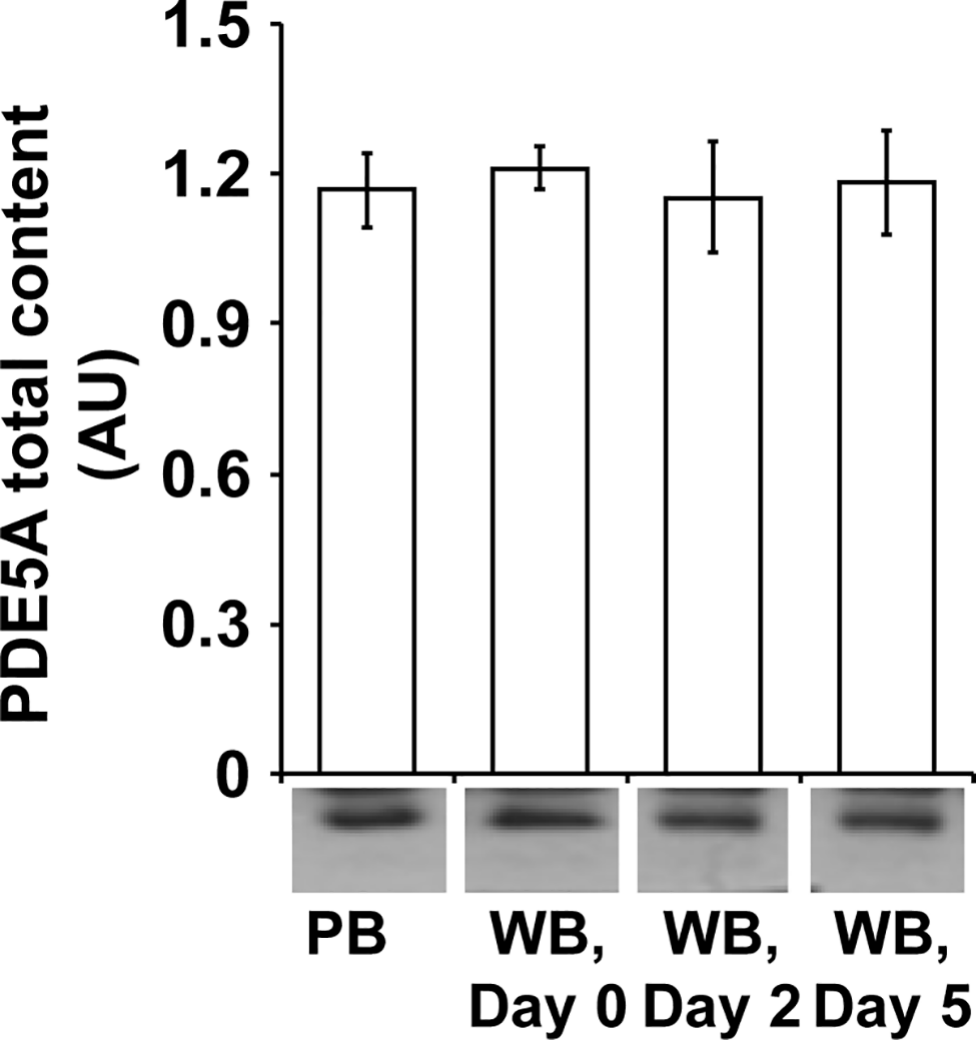
The content of platelet PDE5A remains stable in citrated WB over the storage period of five days. Quiescent washed platelets (1 x 10^8^ per mL) from PB samples and from stored WB units were lysed with Laemmli buffer and analyzed by Western blot for total PDE5A content. After scanning bands were quantified by the Image J program. The data are presented in arbitrary units (AU) normalized to actin as mean ± SEM; n = 8.

Except for slight fluctuations, both cGMP and cAMP levels were unaffected in WB units until day 5 compared to PB samples (Fig 5C and 5D). Accordingly, the platelet content of PDE5A remained unchanged during storage (Fig 6).

## Discussion

Citrated WB is widely used as a source of platelets for experimental research or for therapeutic purposes in transfusion medicine [3–5]. The stability of platelet integrity in the milieu of citrated WB, however, has not been thoroughly investigated yet. In our study, we analyzed platelets stored in WB units for 5 days at ambient temperature and focused on ADP-mediated responsiveness, as a major functional feature of platelets.

As a possible restriction of the study, it should be considered that platelet integrity may be essentially influenced by the storage milieu. Variations of anticoagulant contents or of temperature may lead to different results. However, storage of WB was performed under defined conditions, representing blood banking standards, with a CPD solution for WB storage. Ambient temperature and the use of CPD as anticoagulant have been proved to result in superior platelet yield and recovery in manufacturing of platelet concentrates from WB units [5]. In contrast, storage at lower temperatures has turned out to be associated with clustering of glycoprotein Ib-alpha receptors on the platelet surface [15], and warm temperatures (37°C) lead to elevated metabolism and rapid loss of function [16,17]. CPD contains glucose [18] which is required to maintain platelet viability under storage for several days due to glucose consumption and continued metabolic processes in blood cells [19].

PB samples, for reference, were drawn with a glucose-free citrate solution, frequently used in experimental medicine to obtain fresh platelets. The results from fresh PB samples and from WB on day 0 were congruent. Therefore, we are convinced that this study allows reliable conclusions on ADP-mediated platelet integrity in citrated blood samples.

The basic parameters of WB units showed that the metabolism of blood cells remained preserved during storage, indicated by decreasing pH and glucose concentrations and accompanied by increasing lactate. The slight elevation of potassium levels has also been observed in earlier studies and is most probably caused by discrete hemolysis of red blood cells related to inhibited membrane ATPase or membrane leakages [20].

For analysis of basic platelet characteristics, the degree of pre-activation was determined by basal levels of CD62P expression and fibrinogen binding, two common laboratory markers of platelet activation used for flow cytometry [21,22]. Only on day 5 of storage, CD62P expression was increased by two-fold confirming that pre-activation is induced in stored WB but only to a low level e.g. compared to platelet concentrates [23–26].

The responsiveness to 10 μM TRAP-6 stimulation is well maintained in stored WB and comparable with PB reaching distinct and stable increases of CD62P expression and fibrinogen binding. Preserved fibrinogen binding is also an important condition for effective platelet aggregation as the result of fibrinogen mediated cross-linking of activated platelets [27,28]. TRAP-6 induced aggregation in PRP from WB stored for 5 days reached values in the reference range for healthy individuals with more than 80% [29] pointing to an essentially intact platelet integrity. These characteristics may contribute to the observed beneficial hemostatic effects of WB in patients with acute hemorrhage [5].

The responsiveness to ADP, as a weak physiological activator, was accordingly tested by light transmission aggregometry reaching normal values in PB samples and in WB on day 0. However, on day 2, the platelets from WB developed a partial reduction of aggregation and, on day 5, aggregation was almost abrogated. In order to elucidate the cause for the deterioration of ADP induced aggregation, purinergic receptor expression and function were tested.

ADP mediates its effects on platelets via the two purinergic receptors P2Y1 and P2Y12 [6–8]. Stimulation of the P2X1 receptor causes a rapid calcium influx in platelets that can synergize P2Y1 effects, thereby contributing to mechanisms of ADP induced platelet aggregation [10]. Comparable to PB, the basal and TRAP-6 stimulated expression of all three receptor types measured by flow cytometry were unchanged during storage of WB for 5 days. Studies addressing purinergic receptor expression after stimulation with TRAP-6 have not been reported before, whereas the agonist-induced increase of surface markers or receptors, e.g. like CD62P or thrombin receptor PAR1, is a known manifestation of platelet activation [21,30–32] and may also include purinergic receptors.

The functional activity was tested by the measurement of calcium induced fluorescence after specific stimulation of Fluo-4AM loaded platelets (P2Y1 or P2X1) or by the flow cytometric VASP assay as the gold standard for the P2Y12 receptor [33]. In contrast to P2Y12 or P2X1, the P2Y1 receptor activity continuously decreased during storage, presumably responsible for the corresponding decline of ADP-induced aggregation, especially on day 5. In addition to the impairment of the P2Y1 receptor itself, activation of platelet phospholipase C switched on via receptor stimulation [9,34] and leading to the opening of intracellular calcium stores may also be tampered, an issue that should be addressed in future studies.

Recently, the analysis of tampered ADP responsiveness in washed platelets also revealed the robustness of the P2Y12 receptor, whereas the decline of ADP induced aggregation was particularly caused by disturbed P2Y1 receptor function [12]. Furthermore, the results support the recommendations provided by the manufacturer of the flow cytometric VASP test kit and allowing the use of citrated blood samples stored for up to 48 hours to analyze the P2Y12 receptor function reliably [35].

In addition to activating systems, platelet integrity is also conditioned by inhibitory pathways [36,37]. The degree of VASP phosphorylation is usually regulated by the levels of the intracellular cyclic nucleotides cAMP and cGMP, which activate the cAMP- and cGMP- dependent proteinkinases, PKG and PKA [37,38]. Stimulation of the purinergic receptor P2Y12 is linked to the down-regulation of adenylyl cyclase activity, thereby lowering cAMP levels [9,34]. PKG preferentially phosphorylates Ser^239^ and consecutively Ser^157^, whereas PKA prefers Ser^157^ and thereafter Ser^239^ [38]. Recently, our research group demonstrated for apheresis-derived platelet concentrates that stored platelets develop an impairment of intracellular cGMP regulation with distinct VASP phosphorylation, resulting in exceeding inhibition of agonist-induced aggregation and fibrinogen binding [39,40]. In WB, however, the only detectable deviation of inhibitory pathways was a slight elevation of VASP phosphorylation at Ser^239^ on day 5.

The susceptibility of the P2Y1 receptor function to deterioration has also been observed in stored platelets from apheresis-derived platelet concentrates, associated even with a complete abrogation of 10 μM ADP induced aggregation on day 2 of storage [41]. The more rapid decrease of P2Y1-mediated calcium flux within two days and the additional affection of the cGMP-dependent inhibitory pathways with increased VASP phosphorylation at Ser^239^ and at Ser^157^ [40] may have contributed to the reduced aggregation response in platelets from apheresis-derived concentrates.

In conclusion, platelet responsiveness is well preserved in citrated WB during storage for 5 days characterized by adequate TRAP-6-induced aggregation and activation. ADP-mediated platelet integrity, however, is more susceptible for deterioration associated with the decrease of ADP-induced aggregation and the impairment of purinergic receptor P2Y1 activity, especially under storage for more than two days. These differences of platelet integrity should be kept in mind using platelets from citrated WB for experimentation or for patient treatment. In transfusion medicine, the observed stability of platelet integrity supports conclusions from previous studies characterizing whole blood units, stored for up to 48 h, as an effective blood product for acute haemorrhage, e.g. in the military setting [3–5]. Moreover, WB units after a hold for 48 h at ambient temperature may be an adequate source for manufacturing platelet concentrates, which should be thoroughly addressed in future studies. The current restriction of WB storage to a maximum of 24 h before processing represents a major logistical limitation of blood donation management. Although refrigeration has shown preserved *in vitro* function of stored platelets even up to three weeks [42], it is not an alternative approach, since chilling is accompanied by decreased *in vivo* recovery [15]. In contrast, in vitro data for platelets stored in WB units at ambient temperature beyond 24 h have been limited. Similar to our results, Hughes et al. showed that the aggregation response to different agonists (i.a. 10 μM ADP) is maintained at least for 48 h for platelets stored in WB at 19° C or at 25°C [43]. The minor affection of platelet activation and of inhibitory pathways, as shown in our study for the first time, gives additional evidence that platelet integrity is well preserved after 48 h of storage at ambient temperature in the WB milieu.

## Supporting information

S1 Fig The original Western blot image of results presented in Fig 5A. (PPT)

S2 Fig The original Western blot image of results presented in Fig 5B. (PPT)

S3 Fig The original Western blot image of results presented in Fig 6. (PPT)
