# Supplementary figures and images for: The role of adenosine diphosphate mediated platelet responsiveness for the stability of platelet integrity in citrated whole blood under ex vivo conditions
Source: PLoS One. 2017 Nov 20;12(11):e0188193. doi: 10.1371/journal.pone.0188193 (PMC5695795; doi:10.1371/journal.pone.0188193)

## Slide 1
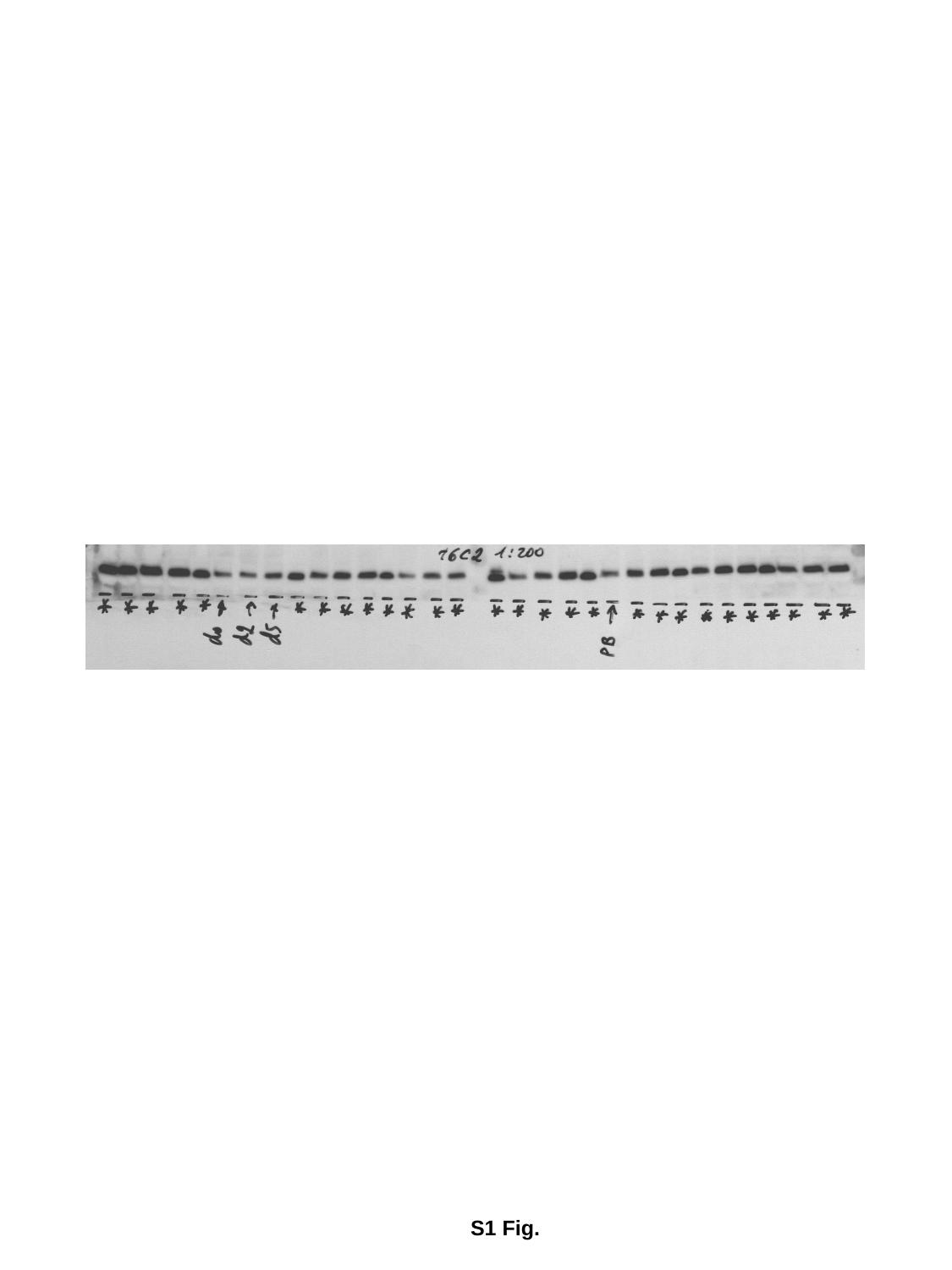

S1 Fig.

Supplement: S1 Fig — (PPT) [file pone.0188193.s001.ppt]

## Slide 1
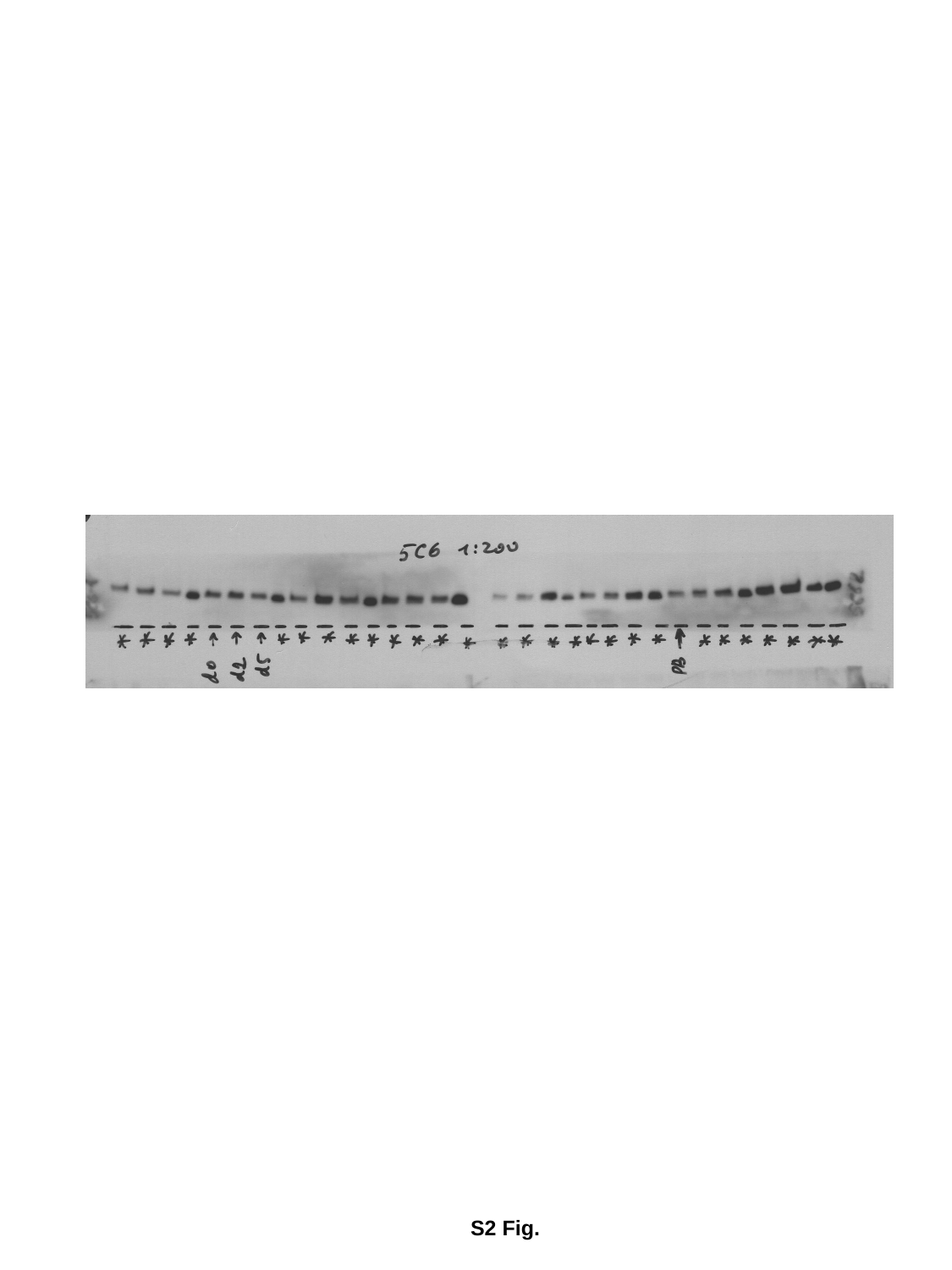

S2 Fig.

Supplement: S2 Fig — (PPT) [file pone.0188193.s002.ppt]

## Slide 1
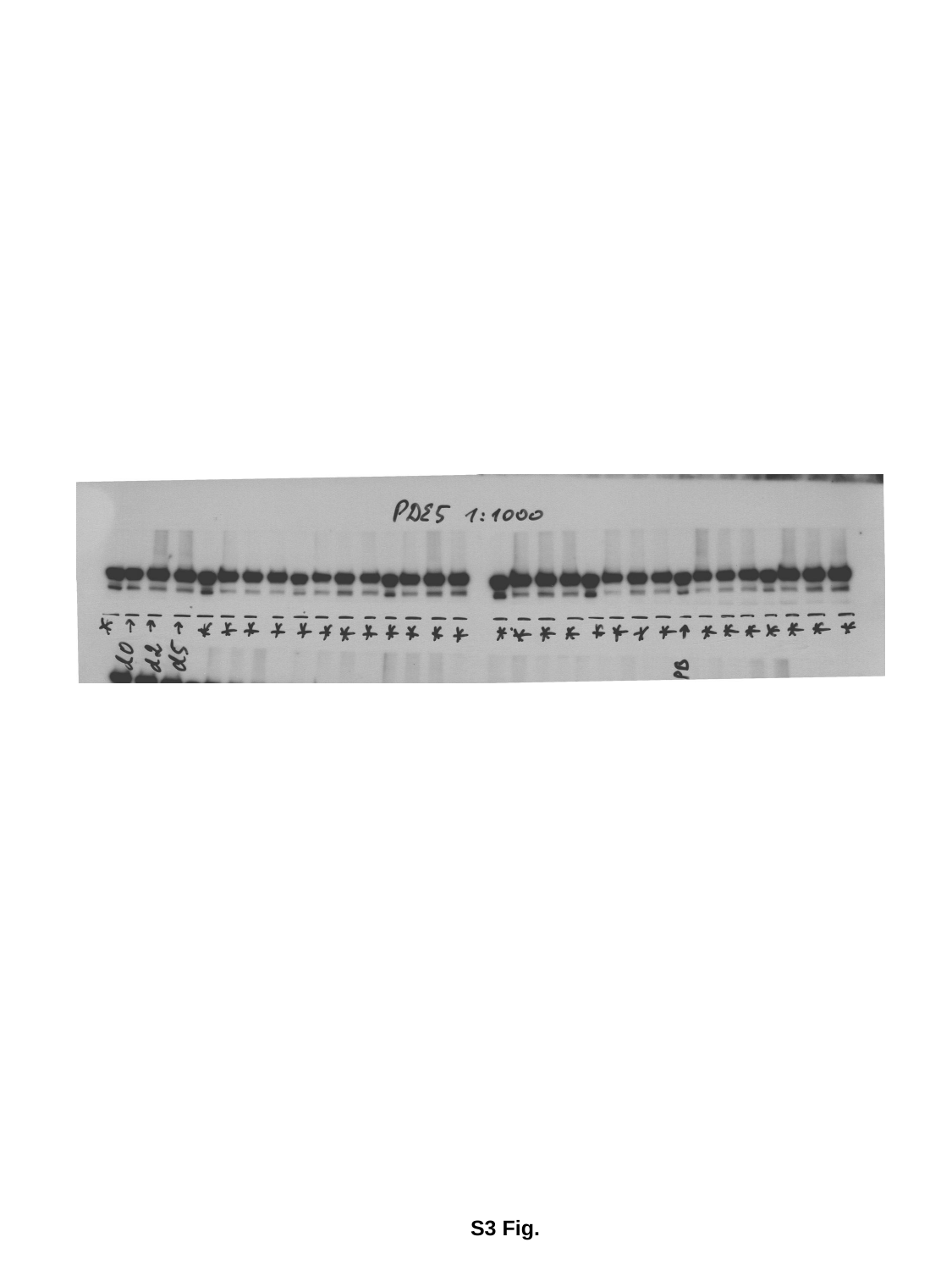

S3 Fig.

Supplement: S3 Fig — (PPT) [file pone.0188193.s003.ppt]
